# Assessment of metals induced histopathological and gene expression changes in different organs of non-diabetic and diabetic rats

**DOI:** 10.1038/s41598-020-62807-0

**Published:** 2020-04-03

**Authors:** Muhammad Ahsan Riaz, Zaib Un Nisa, Muhammad Sohail Anjum, Hira Butt, Azra Mehmood, Ayesha Riaz, Amtul Bari Tabinda Akhtar

**Affiliations:** 1 Department of Environmental Sciences and Engineering, Government College University, Faisalabad, Pakistan; 2 National Centre of Excellence in Molecular Biology, University of the Punjab, Lahore, Pakistan; 3 Department of Zoology, Government College Women University, Faisalabad, Pakistan; 4 Sustainable Development Study Center, Government College University, Lahore, Pakistan

**Keywords:** Biological sciences, Environmental sciences

## Abstract

Diabetes is a complex metabolic disorder and different environmental toxicants including heavy metals have been involved in diabetes induction. Therefore, assessment of the environmental risk factors and heavy metals induced toxicity have become critical for reducing the consequences of metals pollutants. Previously, we reported heavy metals induced nephrotoxicity in non-diabetic and diabetic rats. Here, we extended our analysis by examining the heavy metals induced organs (heart, kidney, liver, pancreas, and spleen) damage in diabetic and non-diabetic Wistar rats using histopathology and quantitative real-time PCR (qRT-PCR). Following the generation of the diabetic rat model, the animals were exposed to heavy metals including lead (Pb), arsenic (As), manganese (Mn) and cadmium (Cd). Both non-diabetic and diabetic rats were exposed to heavy metals for 30 days and subsequently, the heart, kidney, liver, pancreas and spleen tissues were examined. Heavy metal treatment resulted in irregularly arranged myofibrils and vacuolization in the heart tissue of metal treated groups as evident from hematoxylin and eosin (H & E) staining. The kidney tissue of rats treated with heavy metals showed tubular degeneration, fibrosis, hemorrhage, and vacuolation. The liver of the heavy metals treated rats exhibited cellular degeneration and necrosis. The pancreatic tissue of streptozotocin injected untreated and metal treated rats revealed severe degeneration, necrosis, degranulation, shrinkage, and depression in the islets of Langerhans. Increased red pulp area and congestion were observed in the spleen of the metal mixture treated non-diabetic and diabetic rats. In line with the histological data, the qRT-PCR analysis showed downregulated expression of *Bcl*_2_ and upregulation of *Caspase-3* in non-diabetic and diabetic metal treated rats as compared to the non-diabetic untreated rats. In conclusion, the present study revealed, diabetic rats are more prone to metal alone as well as metal mixture induced organ damage as compared to non-diabetic rats.

## Introduction

Diabetes is a metabolic disorder, characterized by impaired insulin secretion, fasting hyperglycemia or insulin receptor insensitivity. The prevalence of diabetes is increasing all over the world and increased from 4.7% in 1980 to 8.5% in 2014^1^. Diabetes is the 7^th^ leading cause of mortality in the United States and worldwide and results in serious complications including kidney disease, cardiovascular disease, blindness, etc.^1,2^. It is well documented that environmental exposure to synthetic or naturally occurring chemical elements contribute to diabetes induction^3–5^. Metals are essential components of biological functions, while their higher concentrations can be toxic^6,7^. Cadmium (Cd), arsenic (As), cobalt (Co), mercury (Hg), manganese (Mn) and lead (Pb) are known as endocrine-disrupting chemicals^8,9^. Importantly, Pb, As, Mn and Cd play a crucial role in public health issues^10^.

Several groups have reported that heavy metals induce toxicity at low concentrations^11,12^. Humans are exposed to heavy metals through water, food or inhalation. Metals readily accumulate in tissues of vital organs via contaminated air, water^13^ and food^14^ resulting in long-term toxic effects. Concentrations of trace elements affect a range of organs in both humans and animals^15^. Heavy metal water and air contamination is a global issue especially in developing countries^16,17^. Pb toxicity in both acute and chronic exposure has the potential to cause many deleterious systematic effects at the cellular level and eventually resulting in different diseases^18^. As, is known as carcinogenic chemical and responsible for toxicity in kidney, lung, skin, urinary bladder, liver and prostate^19^. The Cd toxicity results in physiological damage to transport proteins and mitochondria and causes apoptosis in different organs such as liver, kidney, skin, lungs and reproductive organs^20^. Overexposure to Mn has been reported to generate reactive oxygen species (ROS) and cause extensive neural, cardiovascular and hepatic damage^21^. Research on the toxic effects of individual heavy metal as well as metal mixture under diabetic conditions especially the effects of heavy metals at the molecular level are scarce.

Previously, we reported metal-induced nephrotoxicity in diabetic and non-diabetic Wistar rats^22^. Here, we aimed to investigate the effects of metal alone as well as metal mixture (Pb, Cd, Mn, and As) induced toxicity in different body organs including heart, liver, pancreas, spleen, and kidney at molecular level in diabetic and non-diabetic Wistar rats using histopathology and quantitative real-time PCR (qRT-PCR) analyses.

## Results

**Figure 1:**
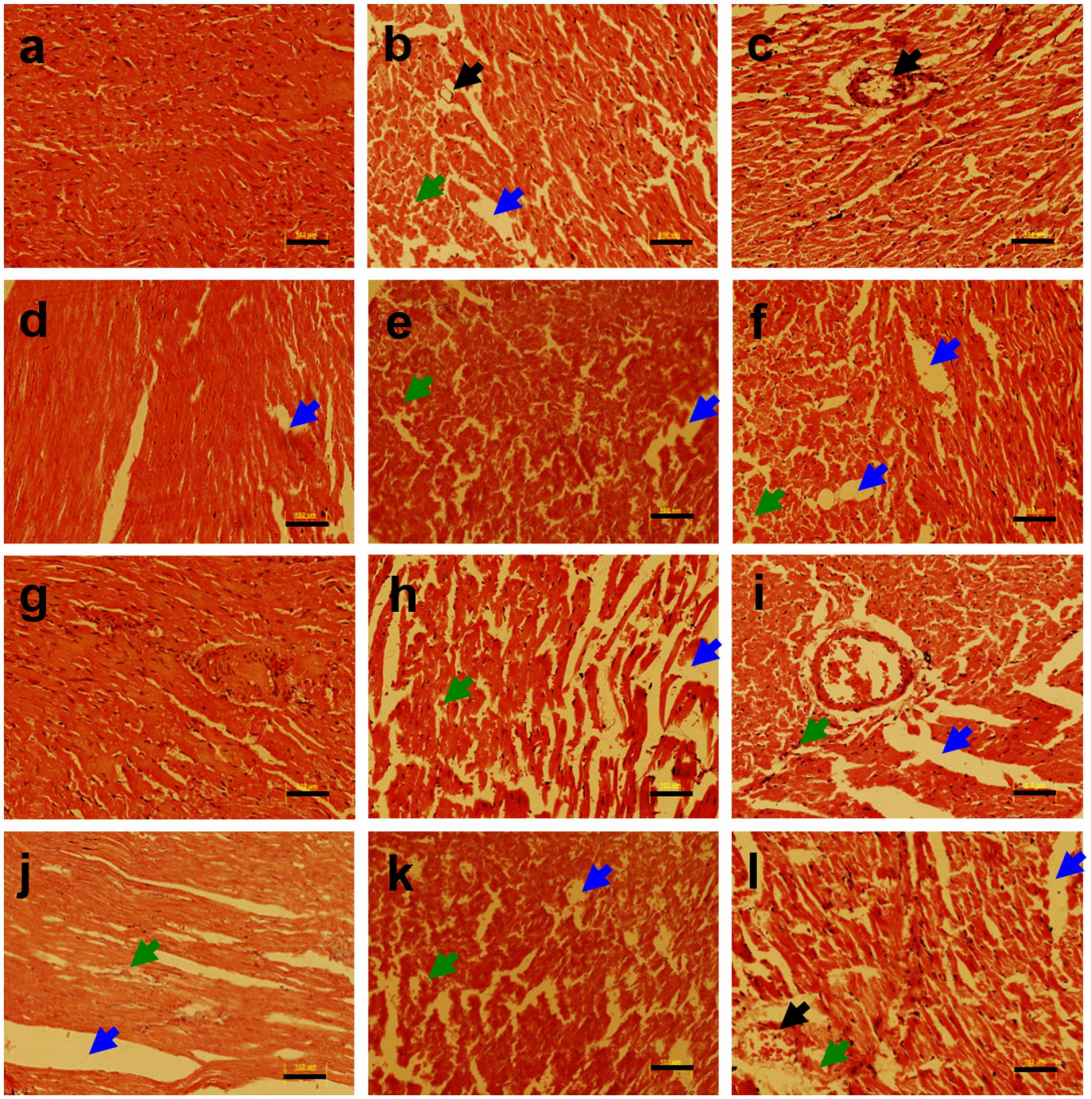
Histopathology of heart tissue of non-diabetic and diabetic rats stained with hematoxylin and eosin (H & E) following heavy metal exposure. (**a**) non-diabetic control group (**b**) non-diabetic arsenic (As)-treated group, (**c**) non-diabetic cadmium (Cd)-treated group, (**d**) non-diabetic manganese (Mn)-treated group, (**e**) non-diabetic lead (Pb)-treated group, (**f**) non-diabetic metal mixture (As + Cd + Mn + Pb)-treated group, (**g**) diabetic untreated group, (**h**) diabetic As-treated group, (**i**) diabetic Cd-treated group, (**j**) diabetic Mn-treated group, (**k**) diabetic Pb-treated group and (**l**) diabetic metal mixture (As + Cd + Mn + Pb)-treated group. Note: All the images represent × 20 magnification and scale bars represent 102 μm. Further, blue arrows represent vacuolation, green arrows represent cellular degeneration and black arrows represent congestion.

Here, we examined the metal alone as well as a metal mixture (Pb, Mn, Cd, and As) induced histopathological changes in different organs (heart, kidney, liver, pancreas, and spleen) of non-diabetic and diabetic Wistar rats. The histological examination of heart tissue from non-diabetic and diabetic rats following heavy metal exposure was performed using a light microscope. To assess the impact of heavy metals on the heart, myocardial tissue was stained with hematoxylin and eosin (H & E). The heart tissue of the non-diabetic untreated control group revealed no histopathological changes including distinct myofibrils arrangement and no congestion in vessels (Fig. 1a). Heavy metal treatment resulted in severe heart damage in the diabetic metal mixture treated group (Fig. 1l). Histological examination of metals treated heart tissue of rat demonstrated irregularly arranged myofibrils and vacuolization (Fig. 1b–l). The effects of heavy metal exposure to non-diabetic and diabetic rats were also evaluated by gene expression analysis for anti-apoptotic and apoptotic markers such as *Bcl*_2_, and *Caspase-3* respectively, using qRT-PCR analysis. To investigate the impact of heavy metals on heart tissue, following RNA extraction and cDNA synthesis, the qRT-PCR analysis was performed. Heavy metal treatment resulted in a downregulated expression of *Bcl*_2_ in non-diabetic and diabetic metal alone as well as in metal mixture treated groups in comparison with the non-diabetic untreated control group as shown in Supplementary Fig. 1a. The qRT-PCR analysis revealed that apoptotic gene *Caspase-3* was significantly upregulated in non-diabetic and diabetic metal alone and metal mixture treated groups in comparison with the non-diabetic untreated control group as shown in Supplementary Fig. 1b.

**Figure 2:**
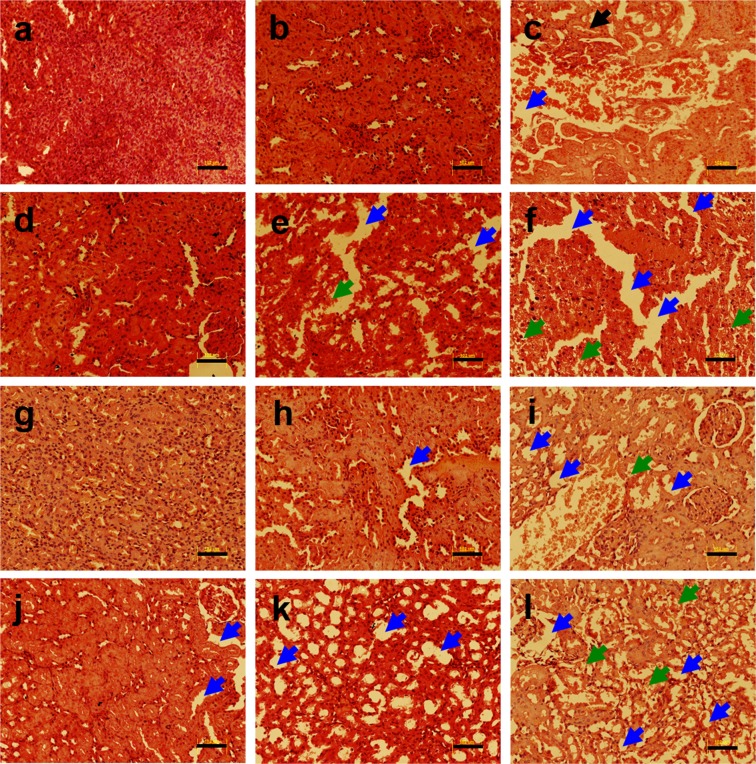
Histopathology of kidney tissue of non-diabetic and diabetic rats stained with hematoxylin and eosin (H & E) following heavy metal exposure. (**a**) non-diabetic control group, (**b**) non-diabetic arsenic (As)-treated group, (**c**) non-diabetic cadmium (Cd)-treated group, (**d**) non-diabetic manganese (Mn)-treated group, (**e**) non-diabetic lead (Pb)-treated group, (**f**) non-diabetic metal mixture (As + Cd + Mn + Pb)-treated group, (**g**) diabetic untreated group, (**h**) diabetic As-treated group, (**i**) diabetic Cd-treated group, (**j**) diabetic Mn-treated group, (**k**) diabetic Pb-treated group and (**l**) diabetic metal mixture (As + Cd + Mn + Pb)-treated group. Note: All the images represent × 20 magnification and scale bars represent 102 μm. Further, blue arrows represent vacuolation, green arrows represent cellular degeneration and black arrows represent congestion.

The histological examination of kidney tissue from the non-diabetic untreated control rats following H & E staining showed normal structure (Fig. 2a), whereas, the kidney tissue from the rats treated with heavy metals exhibited tubular degeneration, fibrosis, hemorrhage and vacuolation (Fig. 2b–l). In Cd-treated, Pb-treated and metal mixture treated non-diabetic as well as diabetic rats, there was an increased severity of pathological changes including red blood cells (RBCs) deposited in capillaries, clogged blood vessels and interstitial fibrosis (Fig. 2c,e,f,i,k,l). In conclusion, exposure to heavy metals resulted in nephrotoxicity in rats. Gene expression analysis of kidney tissue for *Bcl*_2_ and *Caspase-3* following heavy metal treatment revealed downregulated expression of *Bcl*_2_ in non-diabetic and diabetic metal alone and metal mixture treated groups as compared to the non-diabetic untreated control group as shown in Supplementary Fig. 2a. The qRT-PCR analysis revealed that apoptotic gene *Caspase-3* was significantly upregulated in non-diabetic and diabetic metal alone and metal mixture treated groups as compared to the non-diabetic untreated control group as shown in Supplementary Fig. 2b.

**Figure 3:**
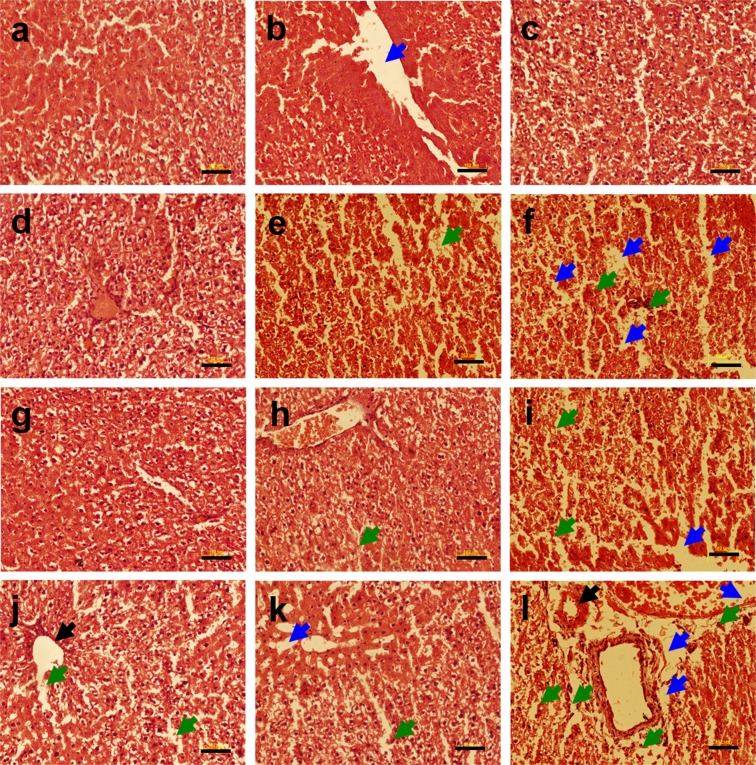
Histopathology of liver tissue of non-diabetic and diabetic rats stained with hematoxylin and eosin (H & E) following heavy metal exposure. (**a**) non-diabetic control group (**b**) non-diabetic arsenic (As)-treated group, (**c**) non-diabetic cadmium (Cd)-treated group, (**d**) non-diabetic manganese (Mn)-treated group, (**e**) non-diabetic lead (Pb)-treated group, (**f**) non-diabetic metal mixture (As + Cd + Mn + Pb)-treated group, (**g**) diabetic untreated group, (**h**) diabetic As-treated group, (**i**) diabetic Cd-treated group, (**j**) diabetic Mn-treated group, (**k**) diabetic Pb-treated group and (**l**) diabetic metal mixture (As + Cd + Mn + Pb)-treated group. Note: All the images represent × 20 magnification and scale bars represent 102 μm. Further, blue arrows represent vacuolation, green arrows represent cellular degeneration and black arrows represent congestion.

The liver tissue of the non-diabetic untreated control group rats showed no histopathological alterations and exhibited normal hepatocyte structure as evident from H & E staining (Fig. 3a). However, liver tissue of heavy metals treated rats had cellular degeneration and necrosis (Fig. 3b–l). Further, metal mixture treated rats showed vacuolation and inflammation in the liver in addition to cellular degeneration (Fig. 3f,l). The effects of heavy metal exposure to non-diabetic and diabetic rats were also evaluated by gene expression analysis for anti-apoptotic and apoptotic markers such as *Bcl*_2_, and *Caspase-3* using qRT-PCR. Heavy metal treatment resulted in a downregulated expression of *Bcl*_2_ in non-diabetic and diabetic metal alone and metal mixture treated groups as compared to the non-diabetic untreated control group as shown in Supplementary Fig. 3a. The qRT-PCR analysis revealed that apoptotic gene *Caspase-3* was significantly upregulated in non-diabetic and diabetic metal alone and metal mixture treated groups as compared to the non-diabetic untreated control group as shown in Supplementary Fig. 3b.

**Figure 4:**
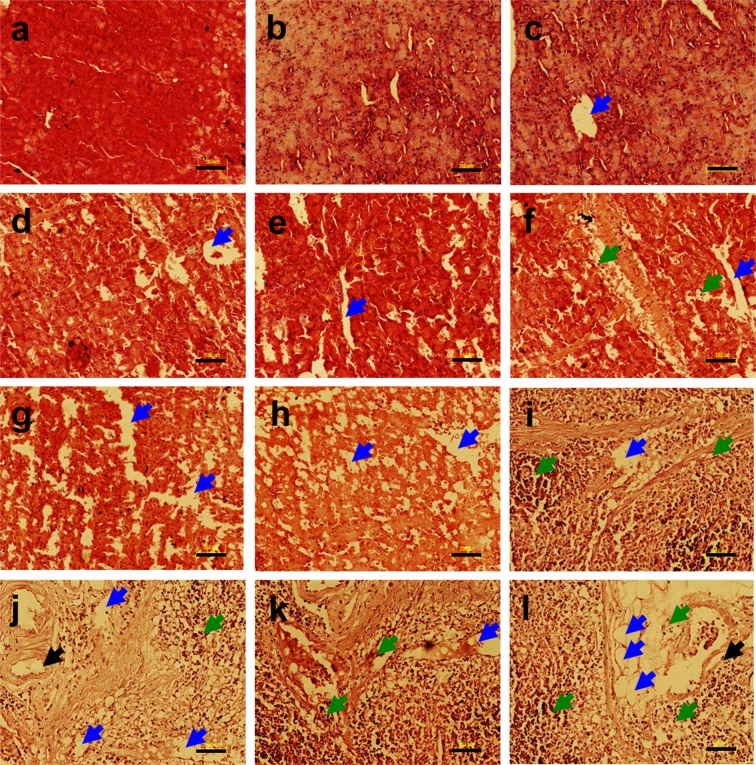
Histopathology of pancreas tissue of non-diabetic and diabetic rats stained with hematoxylin and eosin (H & E) following heavy metal exposure. (**a**) non-diabetic control group, (**b**) non-diabetic arsenic (As)-treated group, (**c**) non-diabetic cadmium (Cd)-treated group, (**d**) non-diabetic manganese (Mn)-treated group, (**e**) non-diabetic lead (Pb)-treated group, (**f**) non-diabetic metal mixture (As + Cd + Mn + Pb)-treated group, (**g**) diabetic untreated group, (**h**) diabetic As-treated group, (**i**) diabetic Cd-treated group, (**j**) diabetic Mn-treated group, (**k**) diabetic Pb-treated group and (**l**) diabetic metal mixture (As + Cd + Mn + Pb)-treated group. Note: All the images represent × 20 magnification and scale bars represent 102 μm. Further, blue arrows represent vacuolation, green arrows represent cellular degeneration and black arrows represent congestion.

The pancreas of the non-diabetic untreated control group rats showed the normal histological morphology (Fig. 4a). In streptozotocin injected untreated rats, severe degeneration and necrosis were observed in pancreatic tissue (Fig. 4g). In diabetic metal treated rats, the most consistent findings in the histologic sections of pancreatic tissues were severe degeneration, necrosis, degranulation and shrinkage in the islets of Langerhans (Fig. 4h–l). The qRT-PCR analysis of pancreas tissue from non-diabetic and diabetic rats following heavy metal treatment exhibited downregulated expression of *Bcl*_2_ in non-diabetic and diabetic metal alone and metal mixture treated groups as compared to the non-diabetic untreated control group as shown in Supplementary Fig. 4a. The qRT-PCR analysis revealed that apoptotic gene *Caspase-3* was significantly upregulated in non-diabetic and diabetic metal alone and metal mixture treated groups as compared to the non-diabetic untreated control group as shown in Supplementary Fig. 4b.

**Figure 5:**
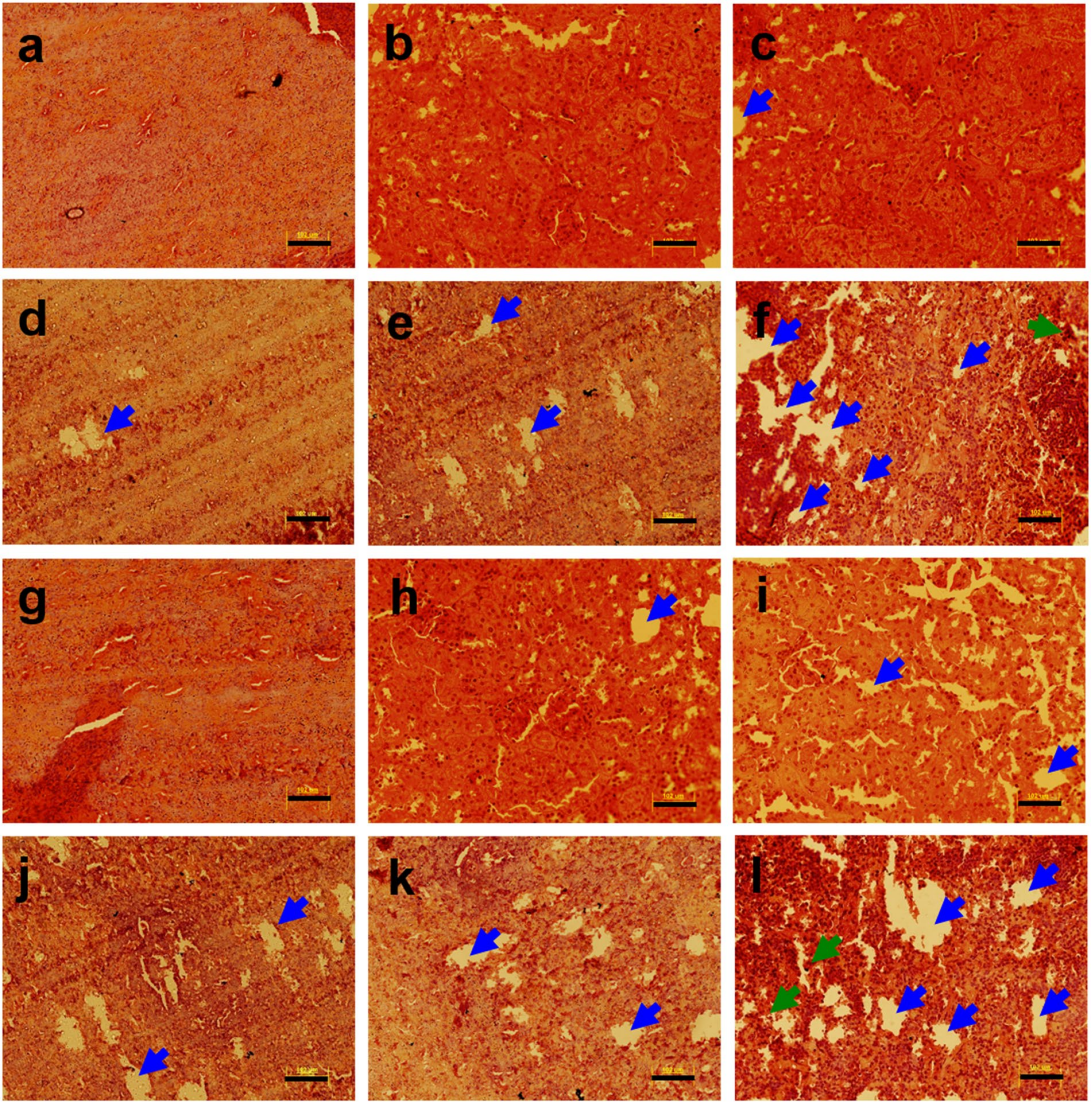
Histopathology of spleen tissue of non-diabetic and diabetic rats stained with hematoxylin and eosin (H & E) following heavy metal exposure. (**a**) non-diabetic control group, (**b**) non-diabetic arsenic (As)-treated group, (**c**) non-diabetic cadmium (Cd)-treated group, (**d**) non-diabetic manganese (Mn)-treated group, (**e**) non-diabetic lead (Pb)-treated group, (**f**) non-diabetic metal mixture (As + Cd + Mn + Pb)-treated group, (**g**) diabetic untreated group, (**h**) diabetic As-treated group, (**i**) diabetic Cd-treated group, (**j**) diabetic Mn-treated group, (**k**) diabetic Pb-treated group and (**l**) diabetic metal mixture (As + Cd + Mn + Pb)-treated group. Note: All the images represent × 20 magnification and scale bars represent 102 μm. Further, blue arrows represent vacuolation, and green arrows represent cellular degeneration.

The images of Fig. 5 show the histopathological sections of the spleen tissue. Severe congestion and enlarged red pulp parts are evident from the spleen tissue of the metal mixture treated non-diabetic and diabetic rats. Severe congestion and necrosis of the spleen tissue are suggestive of heavy metal mixture induced spleen damage (Fig. 5f,l). The effects of metal exposure to non-diabetic and diabetic rats were also assessed by gene expression analysis for anti-apoptotic and apoptotic markers such as *Bcl*_2_, and *Caspase-3* using qRT-PCR analysis. Metal treatment resulted in a downregulated expression of *Bcl*_2_ in non-diabetic and diabetic metal alone and metal mixture treated groups as compared to the non-diabetic untreated control group as shown in Supplementary Fig. 5a. The qRT-PCR analysis revealed that apoptotic gene *Caspase-3* was significantly upregulated in non-diabetic and diabetic metal alone and metal mixture treated groups in comparison with the non-diabetic untreated control group as shown in Supplementary Fig. 5b.

## Discussion

Previously, we reported metal-induced nephrotoxicity using diabetic and non-diabetic Wistar rats^22^. Here, we extended our analysis employing histopathological and qRT-PCR (for *Bcl*_2_ and *Caspase-3* expression) analyses to investigate the metal alone as well as metal mixture induced toxicity in different organs including heart, liver, pancreas, and spleen in addition to the kidney of diabetic and non-diabetic Wistar rats. Diabetes is a complex metabolic disorder^13^ and it is well documented that different toxicants involve in the induction of insulin resistance and contribute to diabetes-related substantial mortalities^23,24^. Apoptosis can be induced by a variety of stimuli including chemotherapy agents^25^, ultraviolet (UV) radiations^26^, polychlorinated biphenyls^27^, infection by pathogens^28^, polycyclic aromatic hydrocarbons (PAHs)^29^ and heavy metals^30,31^. Among these contributing factors, heavy metals take a critical part in the stimulation of apoptosis and eventually leading to cellular death^32^. The Bcl_2_ family includes both pro- and anti-apoptotic molecules^33^. *Caspase-3* is a cytoplasmic pro-enzyme and activate the irreversible process of apoptosis^34^. In the present study, we also examined the expression of *Bcl*_2_ and *Caspase-3* to evaluate the extent of organ damage at the molecular level as a result of heavy metal exposure using qRT-PCR analysis.

Different groups reported the assessment of cardiovascular damage induced by heavy metal exposure using H & E staining^35,36^. Cobbina and colleagues reported high toxicity as a result of co-exposure of metals in comparison with the individual metal exposure^37^. In the present study, the heavy metal treatment caused severe damage in myocardial tissues of non-diabetic, as well as diabetic Pb-treated and metal mixture treated groups exhibiting irregularly arranged myofibrils and vacuolization assessed by H & E staining. Likewise, metal treatment resulted in a downregulated expression of *Bcl*_2_ and up-regulation of *Caspase-3* in non-diabetic and diabetic metal treated groups as compared to the non-diabetic untreated control group. Importantly, Pb and metal mixture treatment resulted in increased cardiac cell apoptosis. Previously, Xu and colleagues reported that Pb induces apoptosis in vascular and cardiac tissues^35^.

Fibrosis and tubular degeneration were observed in kidney tissues of Cd-treated, Pb-treated and heavy metal mixture treated non-diabetic as well as diabetic rats. These findings are consistent with qRT-PCR data that heavy metal treatment resulted in a downregulated expression of *Bcl*_2_ and significant upregulation of *Caspase-3* in non-diabetic and diabetic Pb-treated, Cd-treated, and metal mixture treated groups as compared to the rest of the groups. It is well documented that chronic Cd exposure affects many organ systems and especially kidney has been a critical target of its toxicity^38^. Different groups have reported Pb and Cd-induced renal damage^37,39,40^. Baş and Kalender reported nephrotoxic effects of lead nitrate in diabetic and non-diabetic rats^41^. The cytotoxic effects such as nuclear DNA fragmentation, apoptotic cell production and mitochondrial apoptotic proteins release following CdCl_2_ exposure have been reported in the liver and kidney^42,43^. It has been reported that Pb can induce apoptosis resulting in the imbalance of *Bax*, *Bcl*_2_, and mitochondrial dysfunction^44^. It has been reported that Pb-induced apoptosis may be a probable mechanism of Pb toxicity. Pb exposure induced apoptosis is well known in chicken erythrocytes^45^ and rat proximal tubular cells^46,47^.

The H & E staining of liver tissue showed that heavy metal treatment resulted in cellular degeneration and necrosis in rats. In the liver tissue of metal mixture treated diabetic as well as non-diabetic rats, vacuolation and inflammation were observed in the liver in addition to cellular degeneration. These results are in line with the qRT-PCR data that heavy metal treatment resulted in a downregulated expression of *Bcl*_2_ and upregulation of *Caspase-3* in non-diabetic and diabetic metal alone and metal mixture treated groups as compared to the non-diabetic untreated group. Jayawardena and colleagues reported heavy metals (Cd, Cr, Cu, Pb, and Zn) exposure resulted in histopathological alterations in amphibians^48^. It has also been reported that the administration of Cd cause severe hepatocyte necrosis, fatty changes, degeneration, and inflammatory cell infiltrations^49–51^.

The streptozotocin injected untreated rats revealed severe degeneration and necrosis in pancreatic tissues. In all the heavy metal treated diabetic rat groups, the histologic sections of pancreatic tissues exhibited severe degeneration, necrosis, degranulation and shrinkage in the islets of Langerhans. The qRT-PCR data also confirmed the histology data that metal treatment resulted in downregulated expression of *Bcl*_2_ and upregulation of *Caspase-3* in all the heavy metal-treated diabetic rat groups when compared with their respective non-diabetic heavy metal treated groups revealing the fact that heavy metal treatment might worse the organ damage under diabetic conditions. Kanter and colleagues reported the effects of Cd exposure on morphological aspects of the pancreas, in streptozotocin-induced diabetic rats^52^. Edwards and Prozialeck reported cytotoxic effects of Cd on the pancreatic β-cells^53^. Cd has the potential to induce oxidative stress damage resulting in suppression of insulin secretion and apoptosis in pancreatic islet β-cells *in-vitro* and *in-vivo*^53^.

The present study revealed severe congestion in the spleen tissue of the Cd-treated diabetic rats and metal mixture treated non-diabetic and diabetic rats. Severe congestion in the spleen tissue is suggestive of heavy metal mixture induced spleen damage. As treatment resulted in severe downregulated expression of *Bcl*_2_ and significant upregulation of *Caspase-3* in non-diabetic and diabetic groups as compared to the rest of the groups. In the present study, histological and qRT-PCR data provide an increased understanding of the diabetes dependent damage of lymphoid tissue. Aktug and colleagues evaluated the effects of diabetes on the lymphoid tissue of spleen using immunohistochemistry, qRT-PCR, and light microscope analyses^54^. Zhang and colleagues reported that Cd can induce oxidative stress and apoptosis of spleen by affecting the mitochondrial intrinsic pathway^55^.

In conclusion, the diabetic rats were more prone to metal alone as well as metal mixture induced toxicity as compared to non-diabetic rats. Further, this molecular knowledge may also help in understanding, how different heavy metals individually as well as in combination contribute to organ damage under diabetic and non-diabetic conditions.

## Materials and Methods

### Animals

In the present study, the male Wistar rats (n = 60) having body weight (170–220 grams) were used. We selected male rats for the present study as male individuals working in various foundries (in developing countries) are relatively more exposed to heavy metals as compared to the females and hence males are at high risk as a result of heavy metal exposure. It is also well documented that following streptozotocin treatment, female rats exhibit a more severe form of diabetes with elevated glucose levels, and a worse survival rate as compared to male rats^56^. Another reason to avoid female animals is because of their hormonal fluctuation during the reproductive cycle while males have more stable hormonal status. Because of all the above-mentioned reasons, the male Wister rats were used to study the effects of heavy metals. All experimental protocols were approved by the board of Animal Care, National Centre of Excellence in Molecular Biology, Lahore, Pakistan, and all experiments were performed following approved protocols. Rats were housed at a controlled temperature of (26 ± 1.5 °C), humidity (60% ± 5) and under 12 hours light/dark schedule.

### Development of the diabetic rat model

Type 1 diabetes was induced in male Wistar rats with an intraperitoneal injection of streptozotocin (Sigma Aldrich, USA) at a dose of 40 mg/kg body weight. Serum glucose levels measured on day 6 and rats with glucose level ≥300 mg/dL were considered as diabetic and were used for subsequent heavy metal exposure.

### Heavy metal exposure

The male Wistar rats were divided into the following twelve groups, (1) Non-diabetic control group, (2) Non-diabetic Pb-treated group, (3) Non-diabetic Mn-treated group, (4) Non-diabetic Cd-treated group, (5) Non-diabetic As-treated group, (6) Non-diabetic Pb-, Mn-, Cd-, and As-treated group, (7) Diabetic untreated group, (8) Diabetic Pb-treated group, (9) Diabetic Mn-treated group, (10) Diabetic Cd-treated group, (11) Diabetic As-treated group, (12) Diabetic Pb-, Mn-, Cd- and As-treated group. The Pb, Mn, and Cd were injected intraperitoneally while As was administered orally via drinking water. The name of salts used for heavy metals administration and their concentration are described in Table 1. After heavy metal exposure, every alternate day for 30 days, rats were sacrificed by injecting an overdose of anesthesia followed by cervical dislocation, and then the heart, kidney, liver, pancreas, and spleen were harvested and used for subsequent analyses.

**Table 1:** Details of heavy metal treatments along with the name of salts and their concentration used for heavy metals administration.

Sr. No.	Treatment groups	Salt name	Concentration used
1	Non-diabetic control group	sterile saline solution	Not applicable
2	Non-diabetic Pb-treated group	C_4_H_6_O_4_ Pb	20 mg/kg
3	Non-diabetic Mn-treated group	MnCl_2_	20 mg/kg
4	Non-diabetic Cd-treated group	CdCl_2_	5 mg/kg
5	Non-diabetic As-treated group	AsO_2_Na	60 mg/L
6	Non-diabetic Pb-, Mn-, Cd- and As-treated group	C_4_H_6_O_4_ Pb + MnCl_2_ + CdCl_2_ + AsO_2_Na	20 mg/kg + 20 mg/kg + 5 mg/kg + 60 mg/L respectively
7	Diabetic untreated group	sterile saline solution	Not applicable
8	Diabetic Pb-treated group	C_4_H_6_O_4_ Pb	20 mg/kg
9	Diabetic Mn-treated group	MnCl_2_	20 mg/kg
10	Diabetic Cd-treated group	CdCl_2_	5 mg/kg
11	Diabetic As-treated group	AsO_2_Na	60 mg/L
12	Diabetic Pb-, Mn-, Cd- and As-treated group	C_4_H_6_O_4_ Pb + MnCl_2_ + CdCl_2_ + AsO_2_Na	20 mg/kg + 20 mg/kg + 5 mg/kg + 60 mg/L respectively

Note: C_4_H_6_O_4_Pb (lead acetate), MnCl_2_ (manganese chloride), CdCl_2_ (cadmium chloride) and AsO_2_Na (sodium arsenite).

### Histopathological analysis

The rats were sacrificed and immediately heart, kidney, liver, pancreas and spleen tissues were harvested. Following processing with different grades of ethanol and xylene; liver, kidney, spleen, heart and pancreas tissues were embedded in paraffin wax and stored at 4 °C for further analysis. The tissue sections were made at 5 µm thickness by Microtome (Microm, Germany). Similarly, all tissue sections were mounted on poly-L-lysine-coated microscopic slides. The H & E staining was performed to examine the morphological changes. Following deparaffinization and rehydration, hematoxylin solution was applied on slides for 2 min and then washed in running tap water for 5 min. Subsequently, the eosin solution dropped on sections for 30 sec and washed the slides thoroughly by running tap water. Following dehydration, sections were mounted with cytoseal medium (Richard-Allan Scientific, USA). Four fields per section were randomly selected and photographed with the help of the Olympus BX-61 microscope (Olympus, USA). A minimum of four sections (200 µm apart) selected from each organ per animal (n = 5 rats/group) were used for the study.

### Gene expression profiling

Following sacrificing rats, the heart, kidney, liver, pancreas and spleen tissues were harvested for gene expression analysis. RNA from tissues was extracted using Trizol reagent (Invitrogen, USA) and quantified with an ND-1000 spectrophotometer (NanoDrop Technologies, USA). cDNA was synthesized from 1 µg RNA with the Revert Aid H-Minus first-strand cDNA synthesis kit (Invitrogen, USA) as the manufacturer’s protocol.

The qRT-PCR analysis of all heavy metal treated groups, as well as the non-diabetic untreated control group, was carried out for *Caspase-3* (apoptotic gene) and *Bcl*_2_ (anti-apoptotic gene) using Maxima SYBR Green qPCR Master Mix (Fermentas, USA) according to the manufacturer’s protocol and run on PikoReal 96 real-time PCR (Thermo Scientific, USA). The relative gene expression analysis was performed by using PikoReal software (Thermo Scientific). *β-actin* was used for normalization. The primers were designed using a real-time PCR tool (Integrated DNA Technologies) and are available upon request.

### Statistical analysis

The data were analyzed using the GraphPad Prism 5 software (USA). Results are expressed as mean ± standard deviation (SD). One-way analysis of variance (ANOVA) followed by Dunnett’s post-test was used for analysis. The difference at p ≤ 0.05 considered statistically significant.

## Supplementary information

Supplementary information
