# Supplementary material for: Assessment of metals induced histopathological and gene expression changes in different organs of non-diabetic and diabetic rats
Source: Sci Rep. 2020 Apr 3;10:5897. doi: 10.1038/s41598-020-62807-0 (PMC7125094; doi:10.1038/s41598-020-62807-0)
Supplement: Supplementary file 1 — Supplementary information [file 41598_2020_62807_MOESM1_ESM.pdf]

Title: Assessment of metals induced histopathological and gene expression changes in different organs of non-diabetic and diabetic rats

Muhammad Ahsan Riaz[1*], Zaib Un Nisa[1], Muhammad Sohail Anjum[2], Hira Butt[2], Azra Mehmood[2], Ayesha Riaz[3], Amtul Bari Tabinda Akhtar[4].

[1]Department of Environmental Sciences and Engineering, Government College University Faisalabad, Pakistan.

[2]National Centre of Excellence in Molecular Biology, University of Punjab, Lahore, Pakistan.

[3]Department of Zoology, Government College Women University Faisalabad, Pakistan.

[4]Sustainable Development Study Center Sustainable Development Study Centre, Government College University Lahore, Pakistan.

***Corresponding author:** Muhammad Ahsan Riaz

Corresponding author email: ahsanenv38@gmail.com

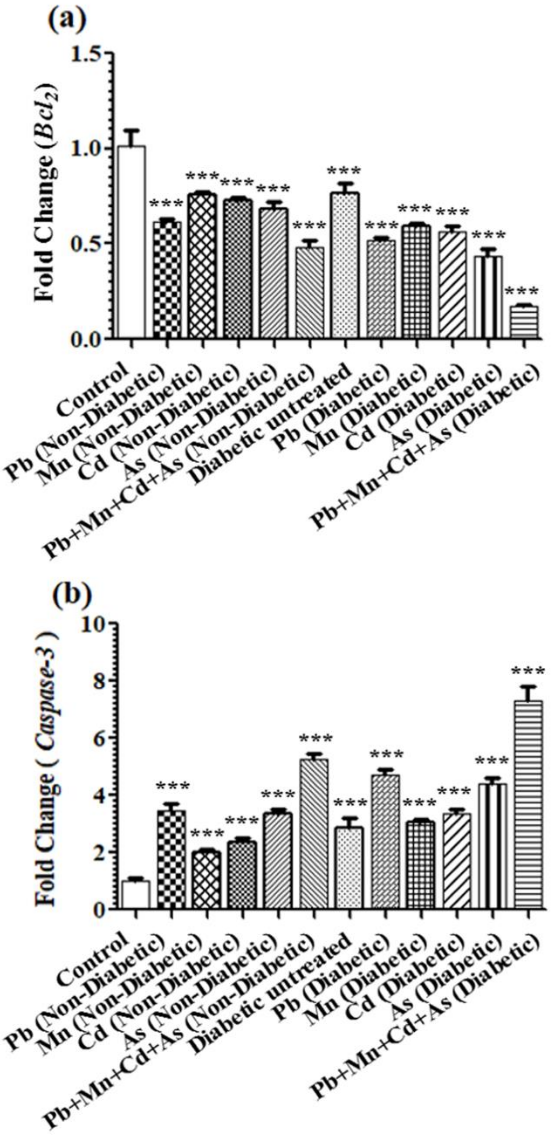

Supplementary Figure 1. Gene expression analysis of heart tissue of non-diabetic and diabetic rats. (**a**) Quantitative real time-PCR (qRT-PCR) analysis of heart tissue following heavy metal exposure for *Bcl*$_2$ and (**b**) qRT-PCR analysis of heart tissue following heavy metal exposure for *Caspase-3*. Expression of *Bcl*$_2$ and *Caspase-3* are normalized against *β-actin*, and all values are relative to their respective expression of the non-diabetic control group. All data are expressed as means ± standard deviation (SD) from measurements on 5 rats for each tissue. ***$p \leq 0.05$ in all heavy metals treated groups and diabetic untreated group versus non-diabetic untreated control group.

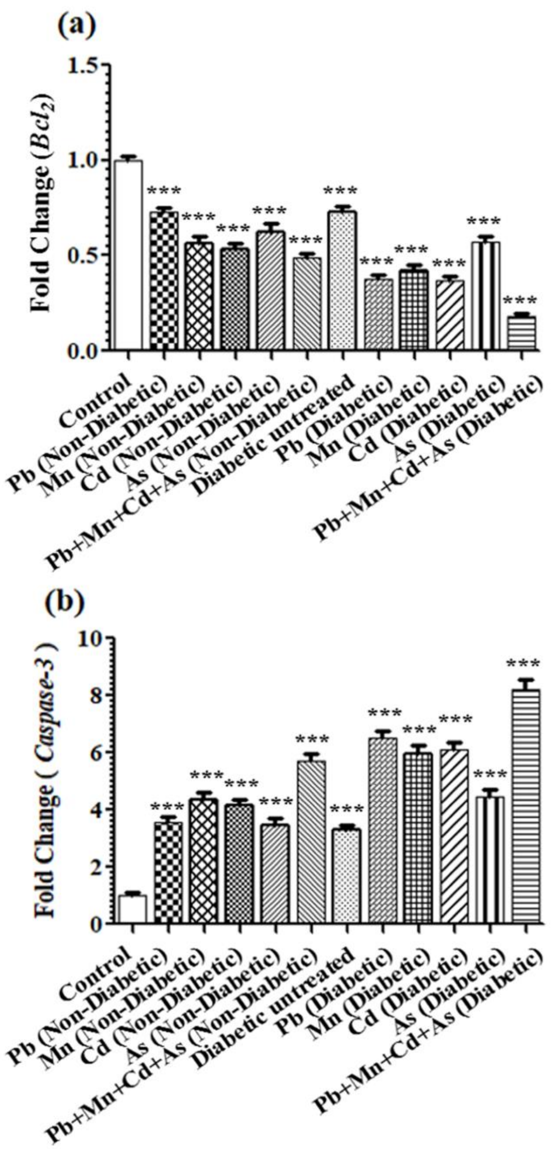

Supplementary Figure 2. Gene expression analysis of kidney tissue of non-diabetic and diabetic rats. (**a**) Quantitative real time-PCR (qRT-PCR) analysis of kidney tissue following heavy metal exposure for *Bcl*$_2$ and (**b**) qRT-PCR analysis of kidney tissue following heavy metal exposure for *Caspase-3*. Expression of *Bcl*$_2$ and *Caspase-3* are normalized against *β-actin*, and all values are relative to their respective expression of the non-diabetic control group. All data are expressed as means ± standard deviation (SD) from measurements on 5 rats for each tissue. ***$p \leq 0.05$ in all heavy metals treated groups and diabetic untreated group versus non-diabetic untreated control group.

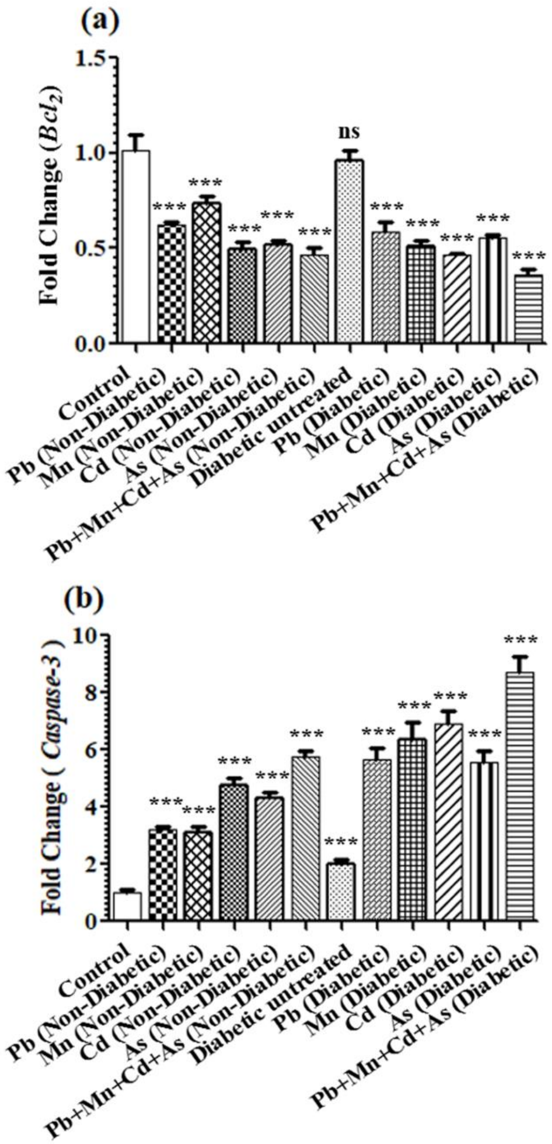

Supplementary Figure 3. Gene expression analysis of liver tissue of non-diabetic and diabetic rats. (**a**) Quantitative real time-PCR (qRT-PCR) analysis of liver tissue following heavy metal exposure for *Bcl*$_2$ and (**b**) qRT-PCR analysis of liver tissue following heavy metal exposure for *Caspase-3*. Expression of *Bcl*$_2$ and *Caspase-3* are normalized against *β-actin*, and all values are relative to their respective expression of the non-diabetic control group. All data are expressed as means ± standard deviation (SD) from measurements on 5 rats for each tissue. ***$p \leq 0.05$ in all heavy metals treated groups and diabetic untreated group versus non-diabetic untreated control group.

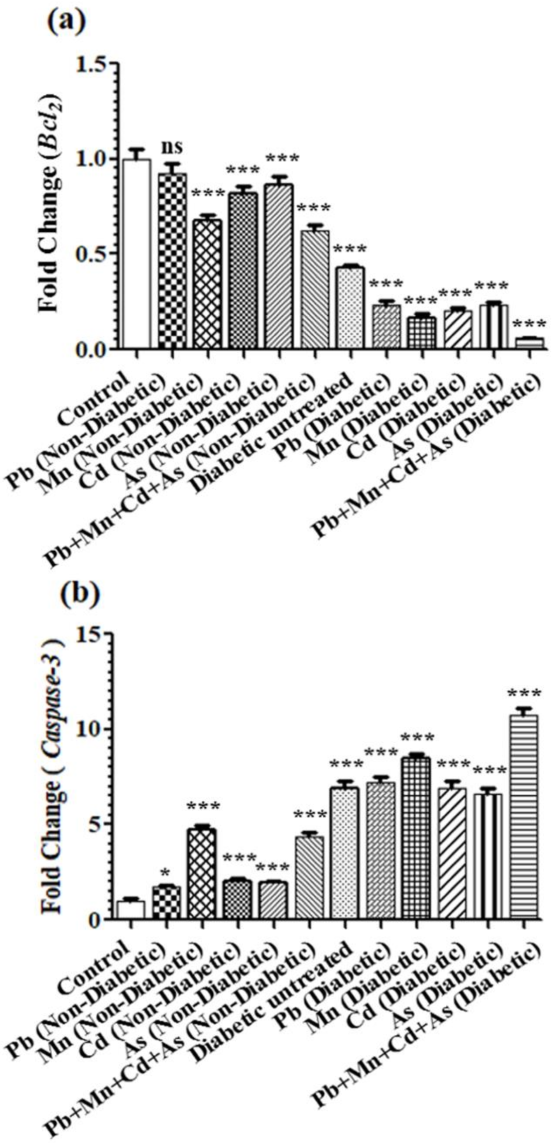

Supplementary Figure 4. Gene expression analysis of pancreas tissue of non-diabetic and diabetic rats. (a) Quantitative real time-PCR (qRT-PCR) analysis of pancreas tissue following heavy metal exposure for *Bcl_2* and (b) qRT-PCR analysis of pancreas tissue following heavy metal exposure for *Caspase-3*. Expression of *Bcl_2* and *Caspase-3* are normalized against *β-actin*, and all values are relative to their respective expression of the non-diabetic control group. All data are expressed as means ± standard deviation (SD) from measurements on 5 rats for each tissue. *, ***$p \leq 0.05$ in all heavy metals treated groups and diabetic untreated group versus non-diabetic untreated control group.

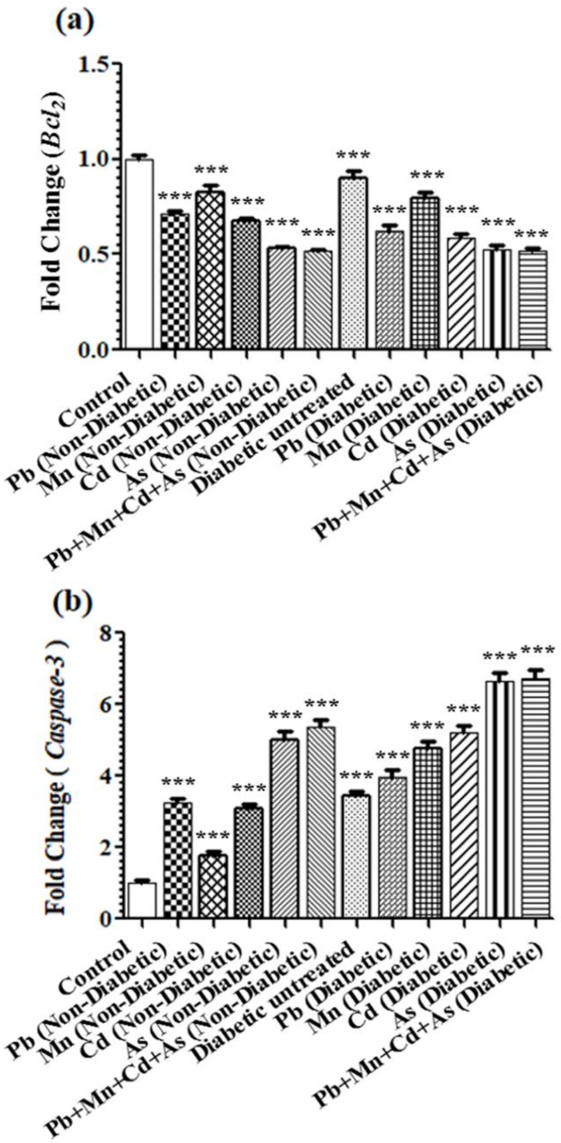

Supplementary Figure 5. Gene expression analysis of spleen tissue of non-diabetic and diabetic rats. (**a**) Quantitative real time-PCR (qRT-PCR) analysis of spleen tissue following heavy metal exposure for *Bcl*$_2$ and (**b**) qRT-PCR analysis of spleen tissue following heavy metal exposure for *Caspase-3*. Expression of *Bcl*$_2$ and *Caspase-3* are normalized against *β-actin*, and all values are relative to their respective expression of the non-diabetic control group. All data are expressed as means ± standard deviation (SD) from measurements on 5 rats for each tissue. ***$p \leq 0.05$ in all heavy metals treated groups and diabetic untreated group versus non-diabetic untreated control group.
